# Clinical characteristics and prostate-cancer-specific mortality of competitive risk nomogram in the second primary prostate cancer

**DOI:** 10.3389/fonc.2023.918324

**Published:** 2023-05-16

**Authors:** Chaojie Xu, Dongchen Pei, Yi Liu, Jianhua Guo, Nan Liu, Qian Wang, Yang Yu, Zhengjun Kang

**Affiliations:** Department of Urology, the Fifth Affiliated Hospital of Zhengzhou University, Zhengzhou University, Zhengzhou, Henan, China

**Keywords:** second primary malignancy, SEER database, competing risk regression analysis, nomogram, prostate cancer-specific mortality (PSCM)

## Abstract

**Background:**

With the development of early diagnosis and treatment, the second primary malignancy (SPM) attracts increasing attention. The second primary prostate cancer (spPCa) is an important class of SPM, but remains poorly understood.

**Methods:**

We retrospectively analyzed 3,322 patients with spPCa diagnosed between 2004 and 2015 in the Surveillance, Epidemiology, and End Results (SEER) database. Chi-square test was applied to compare demographic and clinical variables and analyze causes of death. Multivariate competitive risk regression model was used to identify risk factors associated with prostate-cancer-specific mortality (PCSM), and these factors were enrolled to build a nomogram of competitive risk. The C-index, calibration curve, and decision curve analysis (DCA) were employed to evaluate the discrimination ability of our nomogram.

**Results:**

The median follow-up (interquartile range, IQR) time was 47 (24–75) months, and the median (IQR) diagnosis interval between the first primary cancer (FPC) and spPCa was 32 (16–57) months. We found that the three most common sites of SPM were the urinary system, digestive system, and skin. Through multivariate competitive risk analysis, we enrolled race (p < 0.05), tumor–node–metastasis (TNM) stage (p < 0.001), Gleason score (p < 0.05), surgery (p = 0.002), and radiotherapy (p = 0.032) to construct the model to predict the outcomes of spPCa. The C-index was 0.856 (95% CI, 0.813–0.899) and 0.905 (95% CI, 0.941–0.868) in the training and validation set, respectively. Moreover, both the calibration curve and DCA illustrated that our nomogram performed well in predicting PCSM.

**Conclusion:**

In conclusion, we identified four risk factors associated with the prognosis of spPCa and construct a competing risk nomogram, which performed well in predicting the 3-, 5-, and 10-year PCSM.

## Introduction

The early screening and improved treatment resulted in the increased number of cancer survivors. Meanwhile, the possibility of people developing second primary malignancy (SPM) increased accordingly (1). According to Warren, the histology of SPM is different from the original primary cancer, and the diagnostic interval time is no less than 6 months (2). The SPM is a new type of cancer that is not a recurrence or metastasis of a primary malignant tumor. Studies have shown that SPM was detected in more than 10% of young cancer patients and approximately 25% of elderly cancer survivors (3, 4). Nowadays, not only extensive literatures have focused on the original primary cancers, but also the SPM attracts increasing attention with the development of early diagnosis and treatment.

Prostate cancer (PCa) is the most common malignant tumor in men (5). In the late 1980s, due to the widely carried out prostate-specific antigen (PSA) screening in the United States, the total incidence of PCa doubled in 6 years (1986–1992) (6). According to the International Agency for Research on Cancer estimates, there were approximately 1.4 million new cases of PCa and 375,000 deaths worldwide in 2020; PCa was the fifth leading cause of cancer death among men in the world, with incidence rates ranging from 6.3/100,000 to 83.4/100,000 in different regions (7).

The second primary prostate cancer spPCA is a critical class of SPM for PCa, considering that it is an essential cause of cancer-related deaths among men in the United States (8). Several studies have explored the risk factors and outcomes of developing PSM in patients with primary PCa cancer (9, 10). However, there are few studies on the spPCa, and the risk factors associated with spPCa remain unclear.

Thus, the aim of this study was to determine the clinical and demographic factors related to survival of spPCa. Based on the sub-distribution risk method, we tried to create a competitive risk nomogram to predict the 3-, 5-, and 10-year PCSM of the spPCa.

## Materials and methods

### Data source

All original data used in this study were extracted from the Surveillance, Epidemiology, and End Results (SEER) database (www.seer.cancer.gov), which is an open access database. The MP-SIR sector of the SEER*Stat version 8.3.8 (Username:18501-Nov2019, http://seer.cancer.gov/seerstat/) was used to extract SPM cases from nine population-based registries (2004–2015), and a total of 6,099 original cases were extracted. The search conditions were as follows: (1) FPC cases diagnosed between 2004 and 2015, (2) patients with the second primary cancer, (3) diagnosis made by positive histology, and (4) patients with complete follow-up and end point. Clinicopathological parameters of interest, including diagnosis date (year), age, race, TNM stage, grade, PSA, Gleason score, site of FPC, histological type of FPC, surgery history of spPCa, radiation history of spPCa, chemotherapy history of spPCa, and marital status, were extracted.

### Data processing and study design

**Figure 1:**
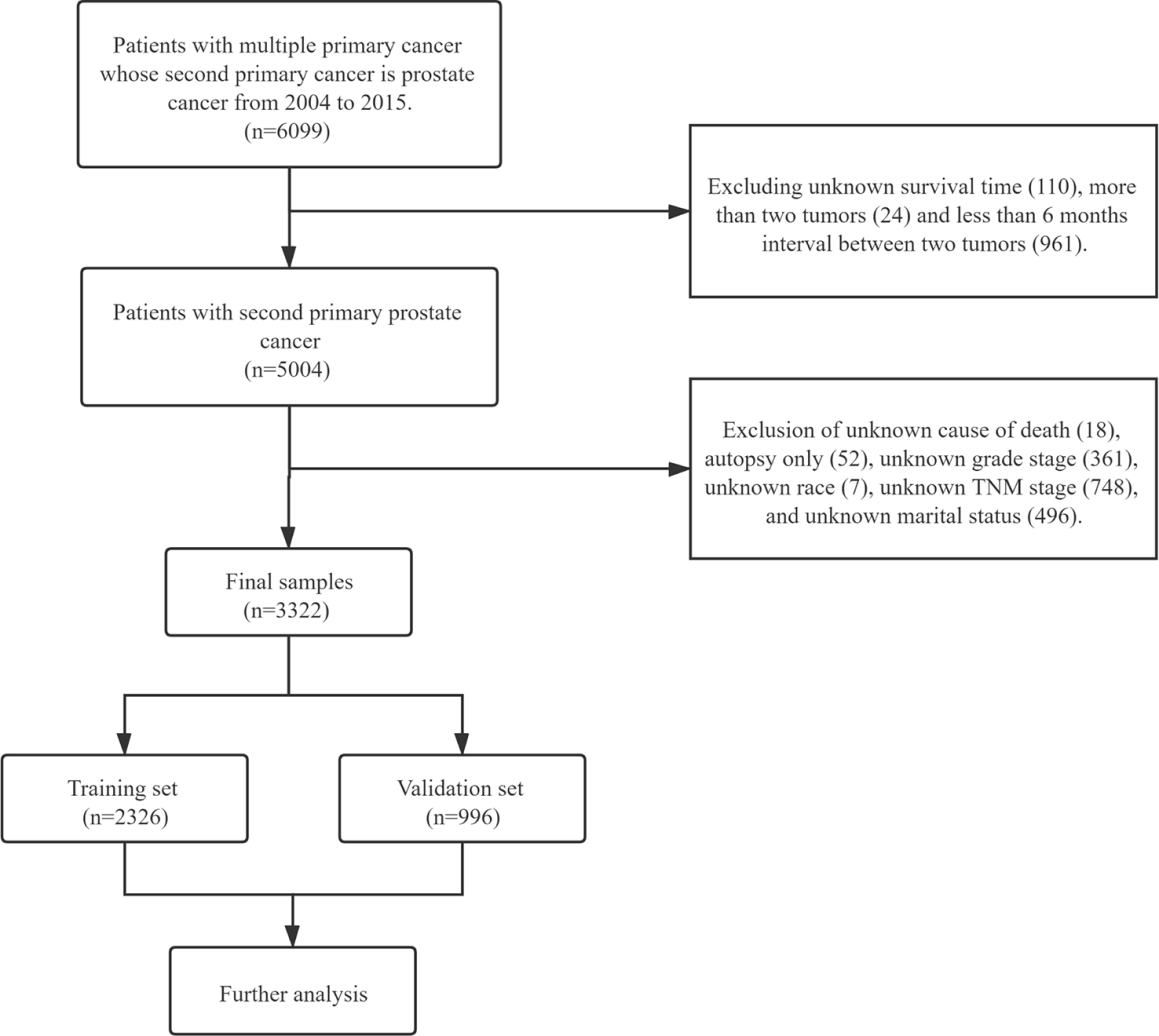
The specific flow chart of case screening in this study.

A total of 3,322 cases were included in this study. The exclusion criteria were listed as follows: (1) the interval between FPC and SPM <6 months (n = 961), (2) patients with only autopsy or death certificate records (n = 52), and (3) patients with unknown clinical information, including no TNM stage of PCa (n =748), unknown marital status (n = 496), no grade of PCa (n=361), unknown survival time of PCa (n = 110), unknown race (n=7), and unknown cause of death (n=18). Patients with more than two primary cancers were excluded (n=24). The detailed flowed chart of patient screening is shown in **Figure 1**.

### Statistical analysis

The normality of the continuous variable’s distribution was checked using Shapiro–Wilk test. Mean ± standard deviation (SD) or median (IQR) was used for continuous variables. Counts and percentages were used for categorical variables. Chi-square test was used for classified variables, and Kruskal–Wallis rank sum test or ANOVA tests were used for continuous variables. Kaplan–Meier (K–M) curves were plotted, and log-rank analysis was applied to compare the overall survival (OS). All cases were randomly divided into training set and validation set at a ratio of 7:3. Then, we incorporated the multivariate competitive risk model and established the competitive risk nomogram to predict the 3-, 5-, and 10-year PCSM of the spPCa. C-index, calibration curve (bootstrap 1,000), and DCA were applied to evaluate the prediction ability and to judge whether the actual result was consistent with the prediction probability.

All statistical analyses were performed in R software (version 3.6.3; http://www.r-project.org/) and EmpowerStats2.0 (www.empowerstats.com). All tests were two-sided, and p < 0.05 was considered statistically significant.

## Results

### Baseline characteristics of study population

After excluding patients with incomplete clinical information, 3,322 eligible patients were enrolled in the study. As shown in **Table 1**, the median year of diagnosis (interquartile range, IQR) of FPC and SPM was in 2007 (2005–2010) and in 2011 (2009–2014), respectively. The median age of diagnosis of FPC and SPM was 65 (59–71) and 68 (62–74) years old, respectively. The median interval between two cancers was 32 (16–57) months. In addition, most registered patients were diagnosed with cancer at the early stage of TNM (I–II, 64.66% of FPC and 86.79% of PCa, respectively). For the treatment of PCa, 41.72% (1,386) of patients had surgery, 35.16% (1,168) had radiotherapy, and only 0.69% (23) had chemotherapy.

**Table 1:** Summary description of demographic and clinical factors.

At prior cancer diagnosis n = 3322		At prostate cancer diagnosis n = 3322	
Variables	Value	Variables	Value
Year of diagnosis		Year of diagnosis	
Median (IQR)	2007 (2005, 2010)	Median (IQR)	2011 (2009, 2014)
Age, year		Age, year	1,778
Mean (SD)	65.00 (9.06)	Mean (SD)	68.33 (8.80)
Median (IQR)	65.00 (59.00, 71.00)	Median (IQR)	68.00 (62.00, 74.00)
Race, n (%)		Race, n (%)	
White	2758 (83.02%)	White	2758 (83.02%)
Black	419 (12.61%)	Black	419 (12.61%)
Other	145 (4.36%)	Other	145 (4.36%)
Marital status, n (%)		Marital status, n (%)	
Married	2291 (75.49%	Married	2489 (74.92%)
Unmarried	744 (24.51%)	Unmarried	833 (25.08%)
TNM stage, n (%)		TNM stage, n (%)	
I-II	2148 (64.66%)	I-II	2883 (86.79%)
III-IV	678 (20.41%)	III-IV	439 (13.21%)
Unknown	496 (14.93%)	Unknown	0 (0.00%)
Survival status		Survival status	
Alive	2548 (76.70%)	Alive	2548 (76.70%)
Dead	774 (23.30%)	Dead	774 (23.30%)
Surgery		Surgery	
Yes	~	Yes	1386 (41.72%)
No	~	No	1936 (58.28%)
Radiotherapy		Radiotherapy	
Yes	~	Yes	1168 (35.16%)
No/Unknown	~	No/Unknown	2154 (64.84%)
Chemotherapy		Chemotherapy	
Yes	~	Yes	23 (0.69%)
No/Unknown	~	No/Unknown	3299 (99.31%)
Interval between diagnoses, months		Time from PCa diagnosis to death or end of study (months)	
Mean (SD)	40.04 (29.28)	Mean (SD)	52.22 (32.52)
Median (IQR)	32.00 (16.00, 57.00)	Median (IQR)	47.00 (24.00, 75.00)

Data were n (%), unless otherwise specified. IQR, interquartile range; PCa, prostatic cancer; SD, standard deviation; ~,Not detectable.

**Figure 2:**
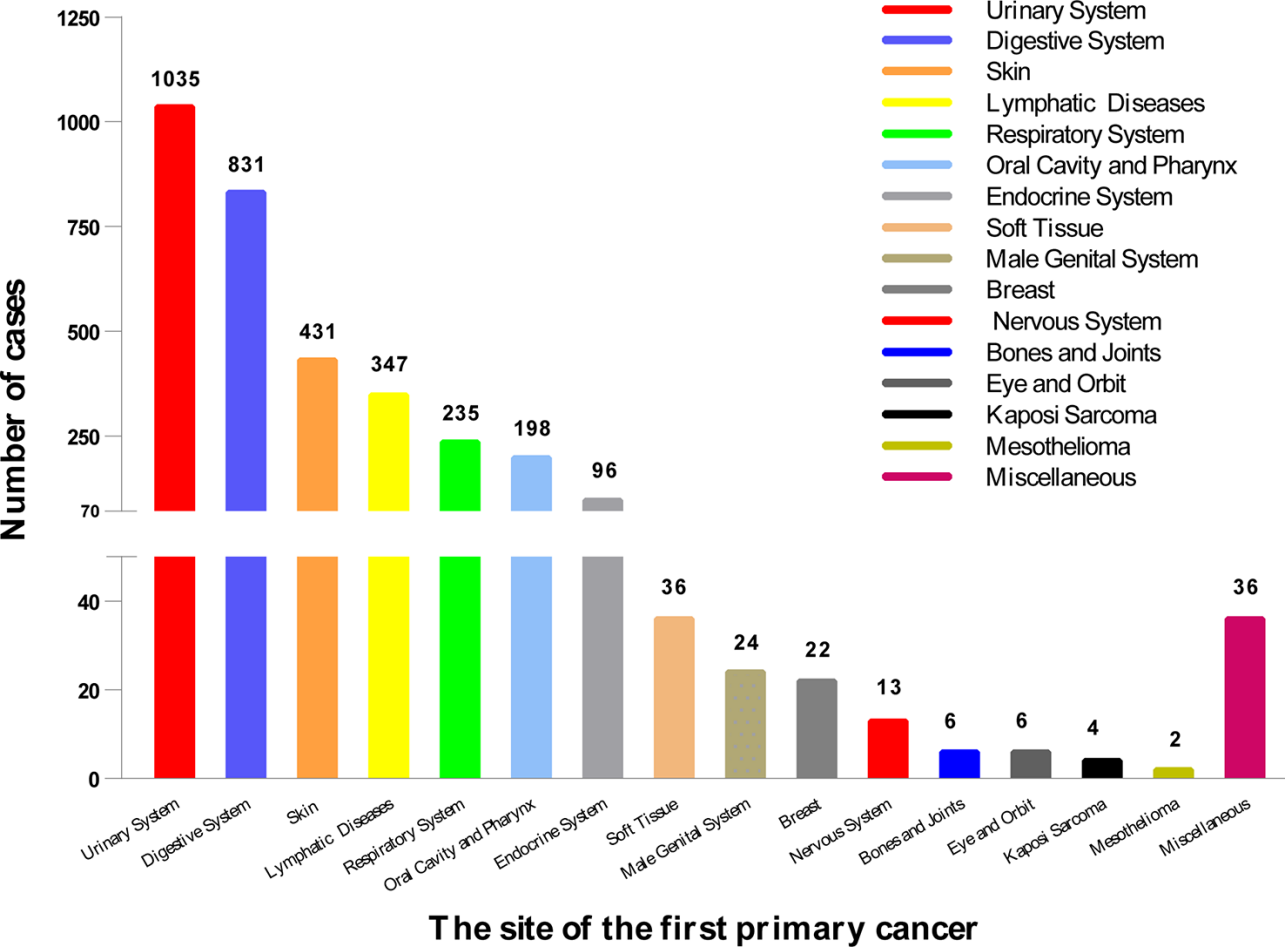
The location of the first primary cancer. We divided it into 16 sites, the most common of which was the urinary system (1,035), followed by the digestive system (831), and the skin (431). The location of 3,322 cases is shown here.

As shown in **Figure 2**, the top 5 common sites of FPC were the urinary system (n = 1,035), digestive system (n = 831), skin (n = 431), lymphatic system (n = 347), and respiratory system (n = 235).

### Mortality of study population

**Figure 3:**
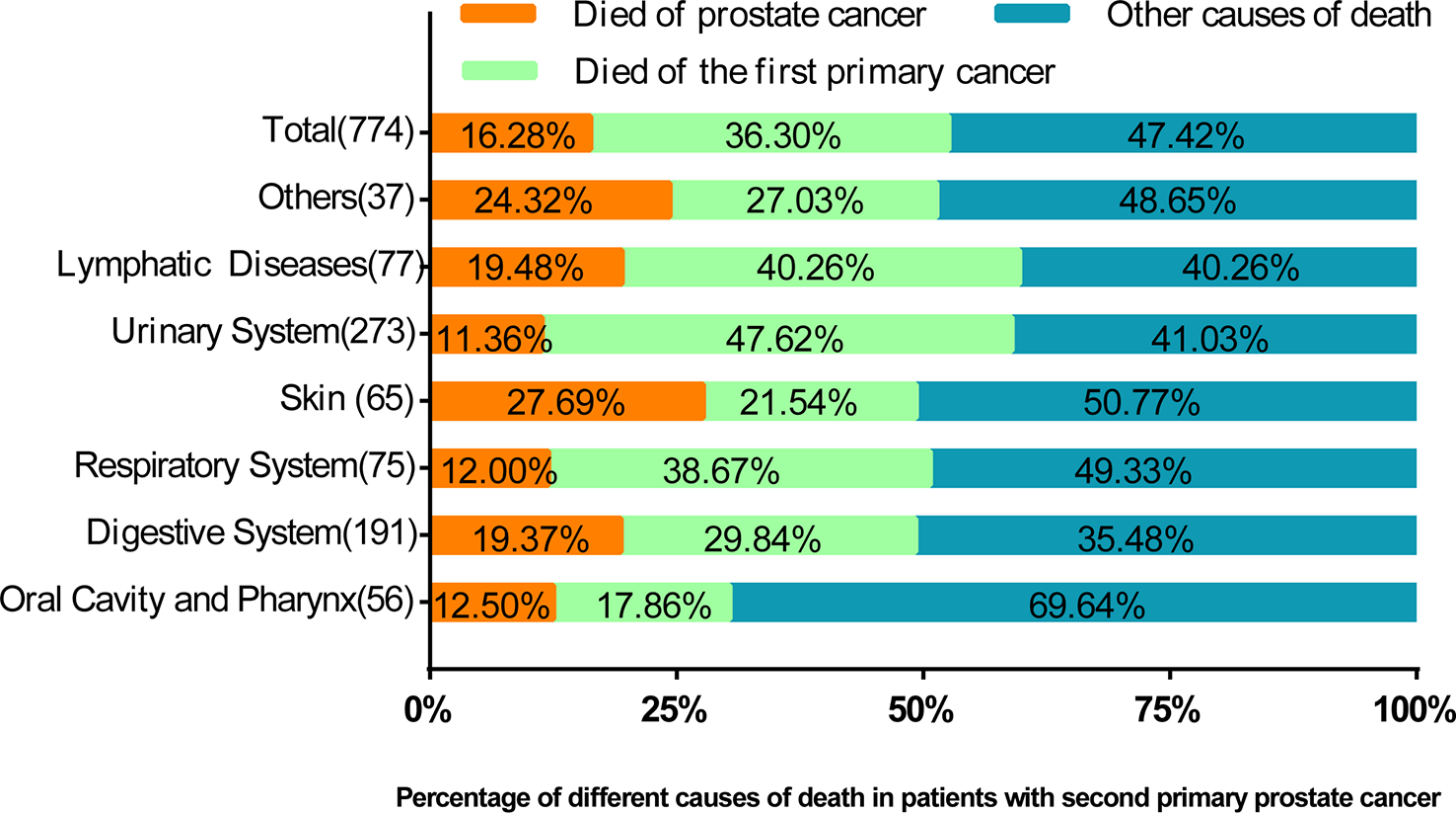
The percentage of prostate cancer deaths, deaths from prior cancer, and other causes of death by the location of the first primary cancer. In this figure, the proportion of causes of death is compared. It can be seen that in most cases, the proportion of deaths caused by prostate cancer is lower than that of previous cancers.

Among 3,322 patients with complete survival data, 23.30% (n = 774) died. We found that 36.30% of the cases died of FPC, 16.28% died of PCa, and 47.42% died of other causes (**Figure 3**). The proportion of patients who died of FPC was higher than that of prostate cancer: lymphatic diseases (40.26% vs. 19.48%, p<0.05), urinary system (47.62% vs. 11.36%, p<0.05), respiratory system (38.67% vs. 12.00%, p<0.05), and oral cavity and pharynx (17.86% vs. 12.50%, p<0.05). The number of deaths caused by PCa in the skin tissue (27.69% vs. 21.54%, p<0.05) was higher than that caused by FPC.

### Kaplan–Meier analysis for prognostic factors

**Figure 4:**
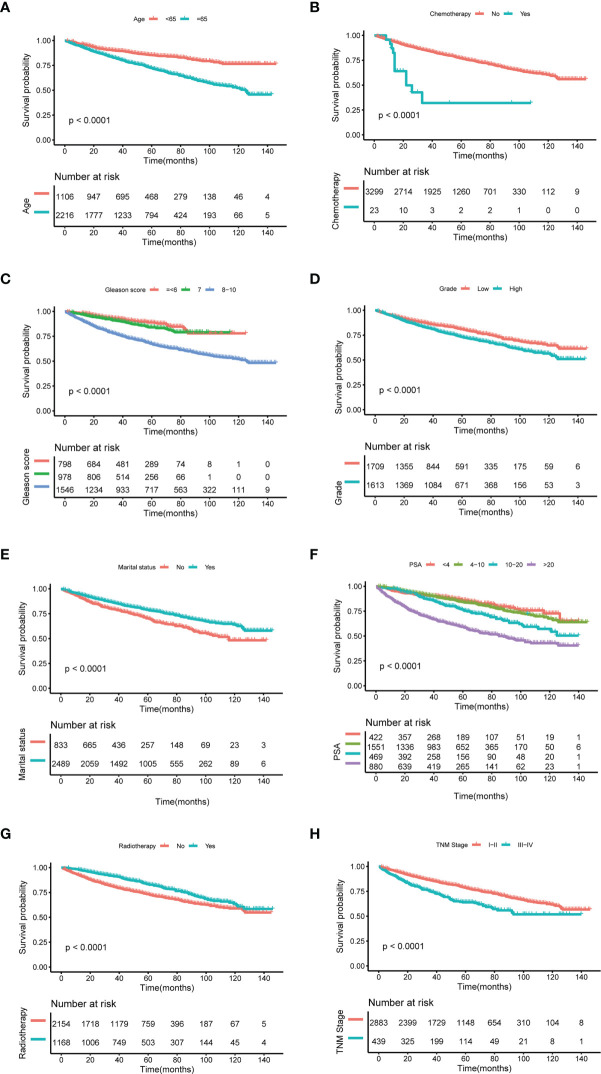
Kaplan–Meier analysis for overall survival (OS) of second primary malignancy (SPM) in the age (p < 0.001) **(A)**, chemotherapy (p < 0.001) **(B)**, Gleason score (p < 0.001) **(C)**, grade (p < 0.001) **(D)**, marital status (p < 0.001) **(E)**, PSA (p < 0.001) **(F)**, radiotherapy (p < 0.001) **(G)**, and TNM stage (p < 0.001) **(H)**.

Kaplan–Meier (K–M) analysis showed that patients with lower age (p < 0.001) (**Figure 4A**), TNM stage (p < 0.001) (**Figure 4H**), PCa grade (p < 0.001) (**Figure 4D**), PSA (p < 0.001) (**Figure 4F**), and Gleason score (p < 0.001) (**Figure 4C**) had prolonged OS. Patients who received radiotherapy for SPM tended to have increased survival (p < 0.001) (**Figure 4G**). While patients who received chemotherapy (**Figure 4B**) or were unmarried (**Figure 4E**) had unfavorable outcomes (p < 0.001).

### Baseline characteristics of training and validation sets

At the ratio of 7:3, 3,322 cases were randomly divided into training set (n = 2,326) and validation set (n = 996). There were no significant differences in age, race, TNM stage, Gleason score, PSA, surgery, radiotherapy, chemotherapy, and marital status (p > 0.05) (**Table 2**). The competitive risk nomogram was constructed within the training set, and the internal validation of the model was carried out. As seen in **Table 2**, most patients were over 65 years old (66.71%), Caucasian (83.02%), and married (74.92%). The majority of the patients had lower TNM stages (stage I–II, 86.79%) and higher Gleason scores (8–10, 46.54%). The number of surgical and unoperated patients was equal (41.72% vs. 58.28%), and most patients did not receive radiotherapy (64.84%) and chemotherapy (99.31%).

**Table 2:** Demographic characteristics of patients with second primary prostate cancer.

	Total (n=3322), n (%)	Training set (n=2326), n (%)	Validation set (n=996), n (%)	*P*
**Age, year**				0.847
<65	1106 (33.29%)	772 (33.19%)	334 (33.53%)	
≥65	2216 (66.71%)	1554 (66.81%)	662 (66.47%)	
**Race**				0.428
White	2758 (83.02%)	1941 (83.45%)	817 (82.03%)	
Black	419 (12.61%)	290 (12.47%)	129 (12.95%)	
Other	145 (4.36%)	95 (4.08%)	50 (5.02%)	
**Marital status**				0.776
Unmarried	833 (25.08%)	580 (24.94%)	253 (25.40%)	
Married	2489 (74.92%)	1746 (75.06%)	743 (74.60%)	
**TNM stage**				0.945
I-II	2883 (86.79%)	2018 (86.76%)	865 (86.85%)	
III-IV	439 (13.21%)	308 (13.24%)	131 (13.15%)	
**Gleason score**				0.058
≤6	798 (24.02%)	577 (24.81%)	221 (22.19%)	
7	978 (29.44%)	658 (28.29%)	320 (32.13%)	
8-10	1546 (46.54%)	1091 (46.90%)	455 (45.68%)	
**PSA, ng/ml**				0.882
≤4	422 (12.70%)	301 (12.94%)	121 (12.15%)	
4-10	1551 (46.69%)	1089 (46.82%)	462 (46.39%)	
10-20	469 (14.12%)	324 (13.93%)	145 (14.56%)	
>20	880 (26.49%)	612 (26.31%)	268 (26.91%)	
**Surgery**				0.676
No	1936 (58.28%)	1361 (58.51%)	575 (57.73%)	
Yes	1386 (41.72%)	965 (41.49%)	421 (42.27%)	
**Radiotherapy**				0.569
No	2154 (64.84%)	1501 (64.53%)	653 (65.56%)	
Yes	1168 (35.16%)	825 (35.47%)	343 (34.44%)	
**Chemotherapy**				0.387
No	3299 (99.31%)	2308 (99.23%)	991 (99.50%)	
Yes	23 (0.69%)	18 (0.77%)	5 (0.50%)	
**Grade stage**				0.477
I-II	1709 (51.44%)	1206 (51.85%)	503 (50.50%)	
III-IV	1613 (48.56%)	1120 (48.15%)	493 (49.50%)	0.847

TNM stage based on 6th edition staging of American Joint Commission on Cancer. PSA, Prostate specific antigen.

### Competing risk analysis

We classified the causes of death as FPC caused, SPM caused, and other caused, and the results of the multivariate competitive risk analysis are displayed in **Table 3**. Race (p < 0.05), TNM stage (p < 0.001), Gleason score (p < 0.05), surgery (p = 0.002), and radiotherapy (p = 0.032) were statistically associated with cancer-specific survival of PCa. PCSM was higher in patients with higher stage (sub-distribution hazard ratio (sdHR), 7.274 (95% CI, 3.704–14.283)). spPCa patients who underwent surgery (sdHR =0.321; 95% CI, 0.155–0.667) had a higher PCSM than those who received no surgical treatment. Additionally, PCSM in patients with radiotherapy (sdHR =0.446; 95% CI, 0.213–0.933) was higher. Black people (sdHR =1.667; 95% CI, 0.711–3.966) and 8–10 Gleason scores (sdHR =1.040; 95% CI, 0.211–5.120) had higher PCSM, although there were no significant differences.

**Table 3:** Competing risk models of probabilities of death from prostate cancer, death from prior cancer, and death from other causes.

Characteristics	Death from prostate cancer		Death from prior cancer		Death from other causes	
	sdHR(95%*CI^2^*)	*P*	sdHR(95%*CI*)	*P*	sdHR(95%*CI*)	*P*
Age, year						
<65	Reference		Reference		Reference	
≥65	1.978(0.762∼5.132)	0.161	1.088(0.746∼1.587)	0.661	2.589(1.681∼3.986)	0.000
Race						
White	Reference		Reference		Reference	
Black	1.667(0.711∼3.906)	0.240	1.622(0.991∼2.655)	0.054	0.718(0.427∼1.209)	0.213
Other	4.526(2.176∼9.414)	0.000	2.165(1.151∼4.071)	0.017	0.393(0.141∼1.096)	0.074
Marital status						
Unmarried	Reference		Reference		Reference	
Married	1.198(0.599∼2.398)	0.610	0.841(0.587∼1.206)	0.346	0.570(0.418∼0.779)	0.000
TNM stage						
I-II	Reference		Reference		Reference	
III-IV	7.274(3.704∼14.283)	0.000	0.254(0.111∼0.580)	0.001	0.718(0.443∼1.165)	0.180
Gleason score						
≤6	Reference		Reference		Reference	
7	0.177(0.032∼0.972)	0.046	1.350(0.611∼2.987)	0.458	1.474(0.849∼2.559)	0.167
8-10	1.040(0.211∼5.120)	0.962	5.194(2.947∼9.154)	0.000	4.039(2.370∼6.882)	0.000
PSA^4^, ng/ml						
≤4	Reference		Reference		Reference	
4-10	1.256(0.265∼5.956)	0.775	0.546(0.305∼0.975)	0.041	0.976(0.553∼1.722)	0.934
10-20	1.574(0.335∼7.401)	0.566	0.756(0.372∼1.534)	0.438	1.025(0.545∼1.926)	0.938
>20	2.331(0.615∼8.841)	0.213	1.785(1.053∼3.027)	0.032	1.068(0.587∼1.942)	0.828
Surgery						
No	Reference		Reference		Reference	
Yes	0.321(0.155∼0.667)	0.002	1.553(0.999∼2.413)	0.050	0.431(0.299∼0.622)	0.000
Radiotherapy						
No	Reference		Reference		Reference	
Yes	0.446(0.213∼0.933)	0.032	0.735(0.437∼1.237)	0.247	0.661(0.459∼0.952)	0.026
Chemotherapy						
No	Reference		Reference		Reference	
Yes	2.436(0.828∼7.173)	0.106	5.326(1.846∼15.368)	0.002	0.291(0.04 0∼2.121)	0.223
Grade stage						
I-II	Reference		Reference		Reference	
III-IV	1.927(0.499∼7.447)	0.341	0.824(0.566∼1.200)	0.312	1.040(0.742∼1.458)	0.817

sdHR, subdistribution hazard ratio; CI, confidence interval; TNM stage based on the 6th edition staging of the American Joint Commission on Cancer (AJCC); PSA, prostate specific antigen.

Similarly, race (p < 0.05), TNM stage (p < 0.001), Gleason score (p < 0.05), PSA (p < 0.05), surgery (p = 0.050), and chemotherapy (p = 0.002) were related to death caused by FPC. People who were non-Caucasian, had I–II TNM stage, high PSA, high Gleason score, and who received surgery and radiotherapy were more likely to die because of FPC. Age (p < 0.001), marital status (p < 0.001), Gleason score (p < 0.05), surgery (p < 0.001), and radiotherapy (p = 0.026) were related to death by other causes.

### Construction and validation of prostate-cancer-specific mortality nomogram

**Figure 5:**
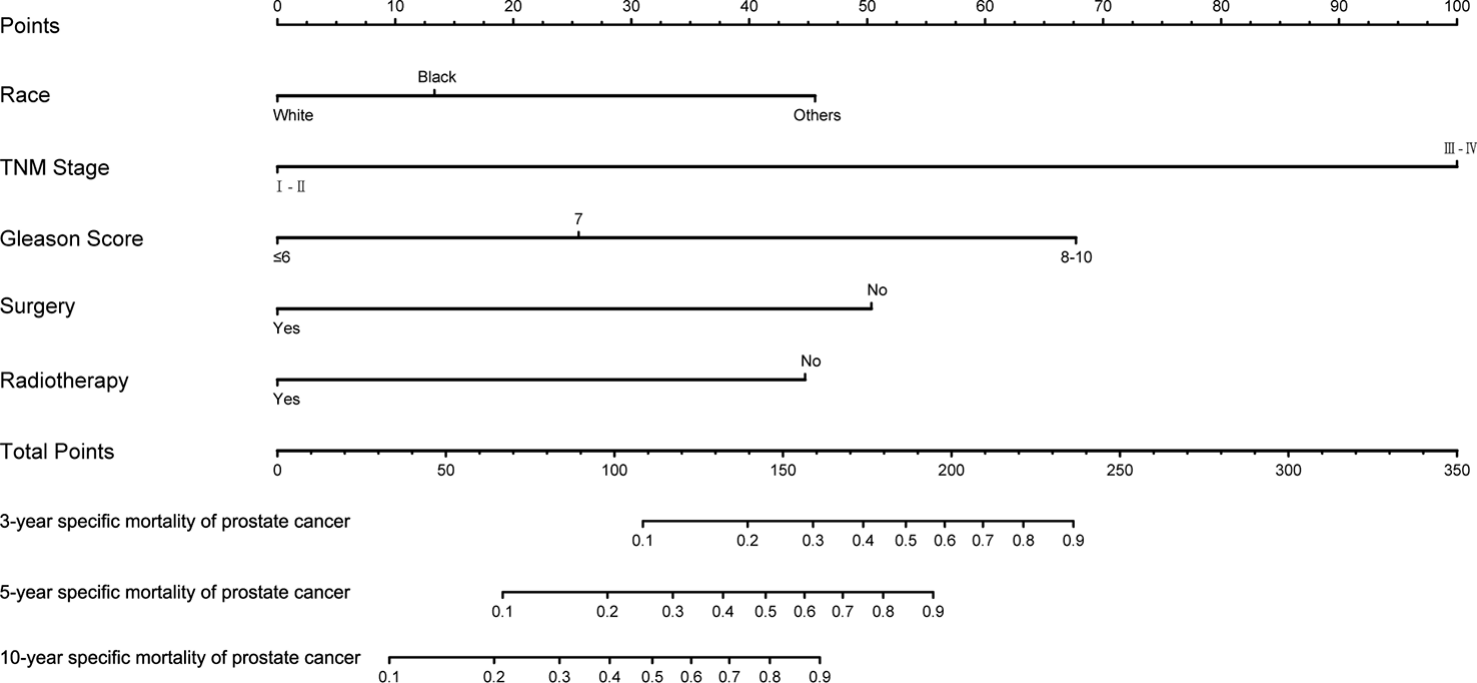
Nomogram to predict 3-, 5-, and 10-year prostate-cancer-specific mortality in patients with second primary prostate cancer.

The training set was analyzed by multivariate competitive risk analysis, and the factors with p < 0.05 were selected to establish a nomogram to predict the 3-, 5-, and 10-year PCSM. Five clinical indicators, including race, Gleason score, TNM stage, surgery, and radiotherapy, were included in our nomogram (**Figure 5**). In order to evaluate the discrimination of the nomogram, we calculated the C-index in the training set (0.856; 95% CI, 0.813–0.899) and the validation set (0.905; 95% CI, 0.941–0.868), which indicated that our model had good distinguishing ability (**Table 4**).

**Table 4:** The discrimination of the nomogram for training set and validation set represented by c-index.

Statistics	Training set	Validation set
C-index (95% CI)	0.856 (0.813–0.899)	0.905 (0.941–0.868)

CI, confidence interval.

**Figure 6:**
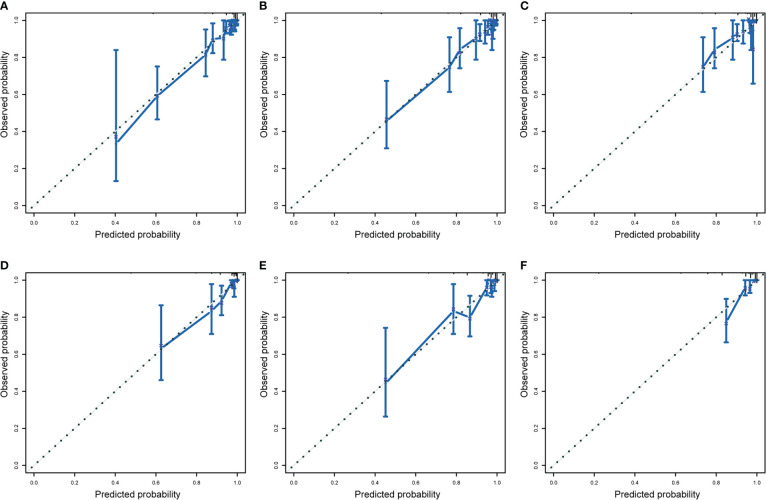
The training set evaluated the calibration curve of 3-year **(A)**, 5-year **(B)**, and 10-year **(C)** prostate cancer-specific mortality (PCSM) in patients with second primary cancer (SPM) and verified the calibration curve to evaluate 3-year **(D)**, 5-year **(E)**, and 10-year **(F)** prostate-cancer-specific mortality in patients with SPM. The dotted line indicates that the prediction probability is equal to the observation probability.

**Figure 7:**
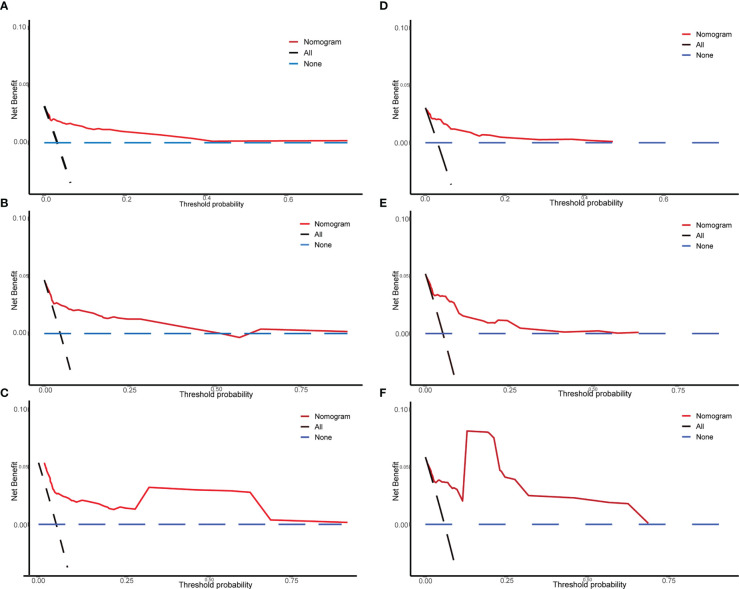
The DCA of the nomogram used in the training and validation sets for predicting prostate-cancer-specific mortality (PCSM). Training set of 3-year **(A)**, 5-year **(B)**, and 10-year **(C)** PCSM of DCA; verification set of 3-year **(D)**, 5-year **(E)**, and 10-year **(F)** PCSM of DCA. The Y-axis represents the net benefit, and the X-axis represents the threshold probability.

We drew calibration graphs of 3-, 5-, and 10-year PCSM in the training and validation sets to judge the consistency of our predictions with actual results (**Figures 6A–F**). Finally, we used the DCA to evaluate the clinical effectiveness of the model (**Figures 7A–F**). Both calibration plot and DCA in the training and validation sets showed good consistency and clinical effectiveness of our model in predicting the PCSM.

## Discussion

It is reported that the number of cancer survivors in the United States is growing at a rate of 2% a year, and according to SEER, approximately 18% of cancer survivors suffer from SPM for the rest of their lives (1). spPCa is a vital subtype of SPM. Therefore, understanding the disease characteristics of spPCa in patients with multiple primary cancer is beneficial to the treatment and prognosis of disease. This study explored the prognosis of the spPCa, identified four risk factors including race, TNM stage, Gleason score, and radiotherapy associated with the prognosis of spPCa, and constructed a competing risk nomogram that performed well in predicting the 3-, 5-, and 10-year PCSM.

The median follow-up time from the diagnosis of spPCa to death or the end of follow-up was 47 months, which was consistent with a study conducted in the United States. Based on the SEER database, it found that 7,852 (7.85%) of 99,977 primary cancer patients developed spPCa, and one in seven men died of spPCa, with an average follow-up period of 66 months (11). Of the 3,322 patients enrolled in our study, 16.28% of people died of spPCa. Compared with patients with only primary PCa, Wu et al. (12) found that only 1.17% patients died of primary PCa, which was much lower than the proportion of PCa deaths (1.17% vs. 16.28%) in our study. In addition, compared with the mortality rate of primary prostate cancer (7.3%) by the International Agency for Research on Cancer estimates, the mortality rate of spPCa patients was significantly worse than that of primary prostate cancer patients. These data strongly suggested that although PCa is generally considered to be an “indolent” cancer, spPCa is still an important cause of death in SPM. Our current results encourage clinicians to treat patients with spPCa.

Our research showed that the five most common sites of SPM were the urinary system, digestive system, skin, lymphatic system, and respiratory system. Similar to our results, previous Swedish studies reported that the most common SPMs were colorectal cancer, skin cancer, bladder cancer, lung cancer, melanoma, and non-Hodgkin’s lymphoma (13). The urinary system being the most common site could be attributed to common carcinogenic pathways, same histological type, chronic inflammatory stimulation, and genetic mutations (14). These results suggested that these vulnerable sites should be carefully monitored.

The treatments of PCa mainly include waiting for active surveillance or watchful waiting (AS/WW), radical prostatectomy, and radical radiotherapy. Generally speaking, the main indications of AS/WW include T1–2, Gleason < 8, low tumor load at biopsy, life expectancy > 10 years, age > 70–75 years, and PSA lower than 10 ng/ml (12). However, a prospective study in the United States pointed out that active monitoring of low-risk PCa might cause patients to miss the best time for treatment, reducing their survival expectations (15). Similar with our results, we found that surgical treatment could reduce the PCSM of spPCa. However, with the development of surgical technology, radical prostatectomy has become the most common treatment for localized PCa (16). Wilt et al. (17) conducted an intervention study on radical prostatectomy and AS/WW for localized PCa. A total of 731 patients with early PCa were enrolled in the group. During 19.5 years of follow-up (median, 12.7 years), death occurred in 223 of 364 men (61.3%) assigned to surgery and in 245 of 367 (66.8%) assigned to AS/WW (absolute difference in risk, 5.5 percentage points; 95% CI, −1.5 to 12.4; hazard ratio, 0.84; 95% CI, 0.70–1.01; p = 0.06). Treatment for disease progression was less frequent with surgery than with AS/WW (absolute difference, 26.2 percentage points; 95% CI, 19.0–32.9). However, it increased the risk of urinary incontinence, erection, and sexual dysfunction within 10 years. Knipper et al. (18) found that 10-year cancer-specific mortality rates were 19.9% vs. 19.6% in radical prostatectomy vs. external beam radiation therapy patients in cumulative incidence smoothed plots. The difference was not statistically significant. This showed that radiation therapy had the same effect as surgery. Similarly, in our multi-factor competitive risk model, the sdHR of radiotherapy was 0.446. Surgery and radiotherapy should be seriously considered for patients with spPCa because it could improve their cancer-specific survival rate.

Due to the lack of relatively accurate evaluation methods, clinicians often made empirical judgments according to the pathological results, TNM stage, medical imaging data, and physical status of patients (19–21). The identified risk factors were limited to advanced age, family history of tumors, and certain gene mutations (e.g., BRCA1 and BRCA2) (22). Through multivariate Cox regression analysis, Liu (10) found that TNM stage (p < 0.001), site of SPM (p < 0.001), and marital status (p = 0.038) were independent prognostic factors for the OS of SPM. However, in SPM, when there were competitive risk events, using traditional survival analysis methods (Kaplan–Meier method, Cox proportional hazard regression model) would overestimate the risk of diseases studied, resulting in competitive risk bias. Some special studies have found that approximately 46% of the literature may have this bias (23), as some survivors usually die of other causes, such as heart disease, before the occurrence of SPM. Therefore, it was suggested that the competitive risk method based on the sub-distributed risk function should be adopted instead of the traditional method (24, 25). As far as we know, some researchers have established a competitive risk model for multiple primary cancers associated with cecal cancer and colon cancer (26, 27), but there was no competitive risk prediction model for patients with spPCa. We have identified that race, TNM stage, Gleason score, and radiotherapy were associated with the prognosis of spPCa using the competing risk model.

As a suitable scoring tool for clinical research, the nomogram could synthesize the influence of a variety of prognostic factors and present the results intuitively (28, 29). In addition, the pros and cons of a predictive model could be measured by distinguishing ability, calibration, and clinical utility (30, 31). It was worth noting that a model with good differentiation may have poor calibration, so both discrimination and calibration were very important to evaluate a model. We calculated the C index of the training set (0.856) and verification set (0.905) and drew calibration curves and DCA. Results showed that our model could better predict the 3-, 5-, and 10-year PCSM with spPCa.

Although we have explored the prognostic risk factors and treatment options and developed a good predictive model for patients with spPCa, there were still some shortcomings in this study. First of all, the SEER database lacked the necessary clinical information and treatment data, such as tumor biomarkers, transcriptome data, immunotherapy, radiotherapy, and chemotherapy dose, and tumor mutation data (32), which made it impossible for us to make a more detailed and comprehensive analysis of the patients. Second, potential bias may have existed when evaluating patients who died of PCa and FPC together, especially when the prevalence of predominantly latent or subclinical PCa rose with age and nearly half of the men aged 50–70 have latent or subclinical PCa. Finally, reasonably designed, large-scale prospective studies are needed to verify the conclusions of this study.

## Conclusion

In conclusion, we identified five risk factors associated with the prognosis of spPCa and constructed a competitive risk nomogram, which performed well in predicting the 3-, 5-, and 10-year PCSM.

## Data availability statement

The raw data supporting the conclusions of this article will be made available by the authors, without undue reservation.

## Ethics statement

The studies involving human participants were reviewed and approved by Ethics Committee of the Fifth Affiliated Hospital of Zhengzhou University. Written informed consent for participation was not required for this study in accordance with the national legislation and the institutional requirements.

## Author contributions

CX, ZK, and DP designed this work. YL, JG, NL and QW integrated and analyzed the data. YY wrote this manuscript. ZK edited and revised the manuscript. All authors approved this manuscript.
